# Nanoparticles in Cancer Diagnosis and Treatment

**DOI:** 10.3390/ma16155354

**Published:** 2023-07-30

**Authors:** Jaya Baranwal, Brajesh Barse, Amalia Di Petrillo, Gianluca Gatto, Luca Pilia, Amit Kumar

**Affiliations:** 1 DBT-ICGEB Centre for Advanced Bioenergy Research, International Centre for Genetic Engineering & Biotechnology, Aruna Asaf Ali Marg, New Delhi 110067, India; 2 US India Business Council|US Chamber of Commerce, DLF Centre, Sansad Marg, New Delhi 110001, India; 3 Department of Medical Sciences and Public Health, University of Cagliari, Monserrato, 09042 Cagliari, Italy; amalia.dip@unica.it; 4 Department of Electrical and Electronic Engineering, University of Cagliari, Via Marengo 2, 09123 Cagliari, Italy; gatto@unica.it; 5 Department of Mechanical, Chemical and Material Engineering, University of Cagliari, Via Marengo 2, 09123 Cagliari, Italy

**Keywords:** nanoparticle, metal nanoparticle, cancer diagnosis, nanotechnology, biomedical

## Abstract

The use of tailored medication delivery in cancer treatment has the potential to increase efficacy while decreasing unfavourable side effects. For researchers looking to improve clinical outcomes, chemotherapy for cancer continues to be the most challenging topic. Cancer is one of the worst illnesses despite the limits of current cancer therapies. New anticancer medications are therefore required to treat cancer. Nanotechnology has revolutionized medical research with new and improved materials for biomedical applications, with a particular focus on therapy and diagnostics. In cancer research, the application of metal nanoparticles as substitute chemotherapy drugs is growing. Metals exhibit inherent or surface-induced anticancer properties, making metallic nanoparticles extremely useful. The development of metal nanoparticles is proceeding rapidly and in many directions, offering alternative therapeutic strategies and improving outcomes for many cancer treatments. This review aimed to present the most commonly used nanoparticles for cancer applications.

## 1. Introduction

Cancer, a multifaceted illness, is becoming a global health crisis and the leading cause of death and disability [1,2]. Around 9.6 million individuals died from cancer in 2018, affecting 18.1 million people worldwide. More than two-thirds of the world’s malignancies can be expected to be diagnosed by the year 2040. About 30% of early deaths in individuals aged 30–69 are brought on by cancer [3]. Utilization of biological agents such as terpenoids, plant alkaloids, anti-metabolites, and DNA-damaging alkylating chemicals are used to treat cancer. Unfortunately, contemporary chemotherapy has several drawbacks, most of which are attributable to the lack of target specificity and defects resulting in deficiencies and inconsistent clinical outcomes. Since normal cells also proliferate rapidly, chemotherapeutics are toxic to normal cells in the bone marrow, macrophages, digestive tract, and hair follicles [4,5]. This results in chronic toxicity, including myelosuppression, thrombocytopenia, anaemia, mucositis, organ malfunction, and alopecia. Due to this, physicians may choose to delay, halt, or modify the dosage of the prescribed treatment [6,7]. In addition to toxicity, chemotherapeutic resistance decreases the effectiveness of anticancer agents [8].

**Figure 1:**
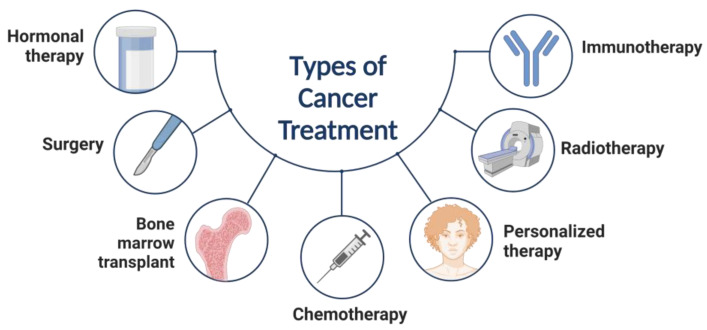
Types of cancer treatment.

Surgical, chemotherapeutic, and radiotherapeutic cancer treatments are now accessible and widely utilized, as seen in Figure 1. However, their target is not just cancer but also healthy cells, which is the most significant problem of modern cancer treatment. Nanoparticles are nontoxic, stable, and biocompatible compounds found in nature, allowing them to be used as an effective drug delivery technique [9].

Metal nanoparticles (NPs) may overcome difficulties associated with traditional treatment. Reportedly, metal NPs perform a positive and potent function in cancer therapy by improving targeting, gene silencing, and medication delivery. Functionalized metal nanoparticles with targeted ligands enhance control over tumor energy deposition in tumors. In addition to their therapeutic use, metal NPs are employed to image cancer cells as a diagnostic tool. Not only do therapeutic systems based on metal NPs give simultaneous diagnosis and treatment, but they also permit regulated and targeted drug release, revolutionizing cancer treatment and control.

Recently, NPs and nanotechnology have drawn a lot of interest in cancer therapy. They receive a lot of attention in the field of cancer therapeutics because they may provide more effective and targeted drug delivery strategies to address the drawbacks of conventional chemotherapy [10,11]. Drug delivery at the target site is hampered by physical and metabolic obstacles [12]. Drug activity is hampered at the cancer level by cellular and non-cellular processes, which increases the risk of recurrence and mortality. Recent decades have seen a substantial increase in scientific interest in nanotechnology because of its distinct functional and physical characteristics [13]. Because of the unique physical and chemical characteristics of NPs, including their chemical composition, small size, vast surface area, and structure, all these applications are feasible and economical [14]. Applications of NPs may be advantageous in treating a number of diseases, including cancer [15]. Attractive potential for NP application includes diagnostics (nanoimaging), drug delivery systems (nanocarrier), and the medical use of NPs themselves [16,17,18].

### Current State of Research on Metal Nanoparticles for Cancer Diagnosis and Treatment

The use of metal nanoparticles in the detection and treatment of cancer has been the focus of extensive investigation. Following is a summary of recent advancements in this area:

*(1). Diagnosis:* Scientists have continued to look at the use of metal nanoparticles as contrast agents for MRI, CT, and optical imaging. The use of lanthanide-doped upconversion nanoparticles, which may transform near-infrared light into visible light and enable deep-tissue imaging, is one recent advance [19]. Researchers have also looked into using metal nanoparticles to identify exosomes, tiny extracellular vesicles released by cancer cells, as a means of early cancer diagnosis. High-sensitivity exosome detection has been achieved by combining gold nanoparticles with surface-enhanced Raman scattering (SERS) [20].

*(2). Treatment:* Metal nanoparticles are still being researched as possible tools for photothermal treatment and localized drug delivery. Utilizing copper nanoparticles as possible therapies has been one recent discovery since it has been demonstrated that they cause cancer cells to die through oxidative stress [21]. Additionally, studies on the utilization of gold nanoparticles as delivery systems for other therapeutics, such as siRNA and chemotherapy medicines, have proceeded [22].

Nanotechnology’s increasingly popular applications in the treatment of cancer are largely due to its distinctively appealing properties for drug delivery, diagnosis, and imaging, the creation of synthetic vaccines, and the development of tiny medical devices, as well as the therapeutic properties of some nanomaterials themselves. Nanotherapies that incorporate some of these characteristics (such as enhanced circulation and reduced toxicity) are already in use, and others are showing great promise in clinical research, with definitive results anticipated in the near future. Several therapeutic nanoparticle (NP) platforms, such as liposomes, albumin NPs, and polymeric micelles, have been approved for the treatment of cancer, and many other nanotechnology-enabled therapeutic modalities, such as chemotherapy, hyperthermia, radiation therapy, gene or RNA interference (RNAi) therapy, and immunotherapy, are under clinical investigation. We have made significant advancements in the field of cancer nanomedicine, but we have also progressively come to understand the difficulties and possibilities that lay ahead. First and foremost, it is obvious that rigorous patient selection is necessary to determine which patients are most likely to benefit from a particular nanotherapy, given the complexity and variety of cancers [23]

Overall, the most recent studies in this area have concentrated on enhancing the specificity and effectiveness of metal nanoparticles for cancer detection and therapy, as well as discovering novel kinds of metal nanoparticles with special features for these purposes.

## 2. Role of Nanoparticles for Cancer, Biomedical Properties, and Therapeutics

**Figure 2:**
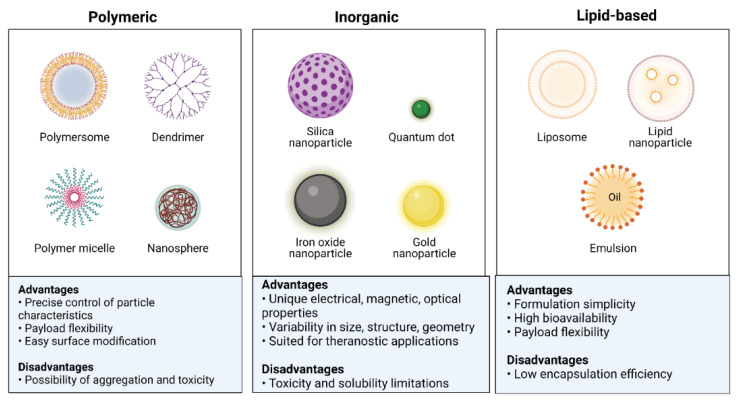
Classification of nanoparticles based on their physicochemical properties.

Nanotechnology has a growing potential for application in medical diagnosis and therapy. Nanotechnology advancements have developed novel and better nanomaterials for biomedical applications [24]. NPs are utilized in various applications due to their unique properties [25]. Multifunctional NPs can transport hydrophobic compounds, target disease cells both actively and passively, extend the time a drug is in the bloodstream, increase the entry and accumulation of pharmaceuticals at tumor sites, overcome drug resistance, increase the safety and tolerability of medications, and advance the development of other technologies [26,27]. NPs are used in medical applications because of their unique properties, such as quantum properties, a surface-to-mass ratio that is much higher than that of other particles, and the ability to absorb and transport other compounds, such as proteins and medicines. NPs can have variable compositions, as their beginning ingredients might be dextran, chitosan, biological lipids, phospholipids, lactic acid, or chemicals such as silica, metals, carbon, and other polymers [28,29,30,31,32]. Classes of NPs, along with their advantages and disadvantages, are shown in Figure 2.

NPs are the perfect theranostic tools for tumor tracking and therapy because of their size (1–100 nm) and the high surface-to-volume ratio [33]. Effective medicine delivery is made possible by directly coupling NPs to various biomolecules, which also permits anatomical and functional imaging. Three distinct processes are involved in the delivery of NPs: (a) systemic localization without RES sequestration; (b) extravasation from intratumoral capillaries; and (c) diffusion and penetration into cancerous cells. NPs are effective anticancer weapons due to the high accumulation levels in tumor cells [34]. Manufacturing NPs bigger than 50 nm prevents RES sequestration [35]. Since solid tumors have a unique microenvironment, NP is desirable for imaging and drug delivery. The tumor microenvironment comprises blood vessels, inflammatory cells, signalling molecules, extracellular matrix, and lymphocytes. Blood vessels, inflammatory cells, signalling molecules, extracellular matrix, and lymphocytes make up the tumor microenvironment [36]. Solid tumors differ from healthy tissue in permeable microvascular environments with capillary pores between 120 and 1200 nm in size, which makes it easier for NP to penetrate tumors [37].

## 3. Metal Nanoparticles and Their Application in Cancer

The use of nanotechnology as a novel technique for cancer diagnosis, monitoring, and treatment has recently attracted interest in the biomedical community. Nanomaterials vary in diameter size from 1 to 1000 nm and display several distinctive features that differ from those seen in tiny particles or bulk materials. Nanomaterials have the potential to be used in several biological applications because of their substantial specific surfaces, high surface activity, robust antioxidant properties, outstanding biocompatibility, and solubility for molecular modifications. Liposomes, carbon nanotubes, polymeric micelles, graphs, quantum dots, metallic NPs, and magnetic NPs are often employed in biomedical applications. Previously, it has been demonstrated that using them extensively can improve treatment results [38].

### 3.1. Gold-Based Nanoparticles

The biological application of metallic nanoparticles, particularly gold nanoparticles (Au NPs), has sparked attention among numerous nanomaterials primarily due to its apparent benefits. Various Au NP forms, including spherical, rod-like, cage-like, and others, in diameters ranging from 1 nm to more than 100 nm, can be prepared rapidly. Au NPs’ form and size significantly impact optical and electrical characteristics [39]. Moreover, Au NPs have a negative charge, and many biomolecules, including genes and targeting ligands, can readily functionalize them [40]. Au NPs are harmless and biocompatible [41]. Surface plasmon resonance (SPR) bands are present in Au NPs, which also have an ultra-small size, a macroscopic quantum tunnelling effect, and a distinct surface effect [42]. Due to these unique characteristics, Au NPs have emerged as the most promising material for various biological applications, such as drug delivery, molecular imaging, and biosensing.

#### Photoacoustic Imaging

Photoacoustic imaging (PAI) is a biomedical imaging technology that employs endogenous and exogenous contrasts and provides insightful data on the cellular and molecular properties of the tissue. Because of their inherent and geometrically induced optical components, Au NPs as exogenous contrast agents hold significant potential for PA imaging. The most popular Au NPs forms for PA imaging include shells, prisms, spheres, rods, cages, stars, and blisters [43].

**Figure 3:**
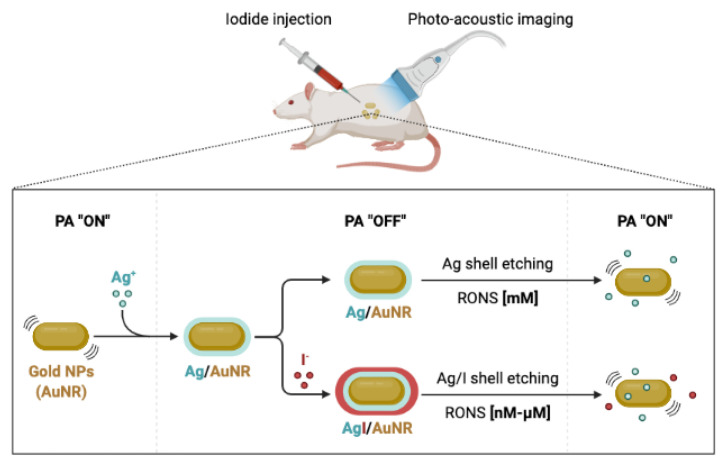
Using iodide-doped gold nanoparticles and photoacoustic imaging to measure oxidative stress (adapted from reference [44]).

Gold nanorods are frequently employed as PAI contrast agents. Using PAI, it has previously been reported that Au/Ag activatable nanoparticles react to reactive oxygen and nitrogen species (RONS). The gold core can be preserved, while just some of the shell can be removed using RONS. The PAI signal is reactivated as a result of the etching. Iodide-doping of silver improves RONS sensitivity, allowing the detection of physiologically relevant levels in a mouse model (Figure 3) [44].

### 3.2. Silver-Based Nanoparticles

Using silver (Ag) NPs as a safe and efficient method for treating cancer is challenging. Integrative studies involving physicists, chemists, engineers for creating NPs, and biologists who can evaluate the effects of Ag NPs in the cell systems they come in contact with, in aspects of cytotoxic effects and ability to damage malignant cells, are required [45]. Ag NPs are important and offer multiple advantages due to their size and shape-dependent features, such as optical, magnetic, chemical, and physical characteristics [46]. Ag NPs are included in numerous items, such as biosensors, composite fibers, antimicrobials, cosmetics, and electrical chemicals [47]. Additionally, Ag NPs can be utilized in cell electrodes, filters, nanocomposites, drug delivery, and medical imaging [47]. Ag is favoured over other NPs due to its superior light absorption, higher resolution, and stronger affinity for functionalization [48].

Like other noble metal NPs, Au NPs display exceptional SPR, making them ideal for usage in various fields, such as biosensing, catalysis, protein/gene transport, and photo-controlled delivery systems [49]. In addition to their antiproliferative effects on cancer cells, silver NPs can activate pathways that inhibit cell division and may be employed in the detection of many cancers like lung cancer, prostate cancer, hepatic cancer, cervical cancer, etc. Research on the antitumor effectiveness of Ag is still actively being conducted, despite technological advancements in its well-defined shapes and sizes and biocompatibility tests of both simple and coated Ag NPs.

### 3.3. Palladium-Based Nanoparticles

The development of palladium (Pd) based NPs is among the innovative nanomaterials that have significantly contributed to advancing the applications of noble metal nanomaterials in biomedicine. Pd-based nanomaterials have distinct advantages over other nanostructures made of noble metals. These advantages include biocompatibility and good photothermal stability. These characteristics make Pd-based nanomaterials stand out as exceptional and promising in biomedicine. After several years of research, Pd-based nanomaterials, such as Pd NSs, Pd@Au, Pd NPs, and Pt@Pt nanostructures, have been the subject of substantial investigation in the field of multimodal imaging-guided cancer treatment.

Pd-based nanomaterials have been noted to exhibit remarkable optical properties, high levels of biocompatibility, and high levels of stability in physiological conditions, all of which make them extremely promising for use in biomedical applications. Research on Pd-based nanomaterials started significantly later than that of other noble nanoparticles that have been extensively studied, such as gold (Au) and silver (Ag) nanomaterials. However, due to distinct qualities, including high photothermal conversion efficiency and photothermal stability, they have attracted a lot of attention in the nanomedicine sector. Pd-based nanomaterials display great near-infrared (NIR) absorption, a fast rate of photothermal conversion, outstanding biocompatibility, and exceptional photothermal stability. Pd nanosheets, porous/hollow Pd NPs, and Pd@M (M = Ag, Au, Pt, SiO_2_, ZIF-8) are examples of these nanomaterials. Pd-based nanoparticles have emerged as promising candidates for use as therapeutic agents and contrast agents in cancer imaging [50,51,52].

Pd nanosheets are typical examples of 2D nanomaterials with powerful NIR absorption, high photothermal conversion efficiency, outstanding photothermal stability, and a high level of biocompatibility. The diameters of Pd nanosheets (Pd NSs) can be easily altered to be anywhere between 5 and 120 nm while maintaining a consistent hexagonal shape [53,54]. The optical absorption peaks of Pd NSs shift depending on their size, but they are always found in the NIR range, thus offering exciting prospects for use in photothermal therapy (PTT). Table 1 summarises cancer therapies that utilize Pd-based nanoparticles, both as stand-alone PTT treatments and in combination with other therapeutic techniques [50].

### 3.4. Iron Oxide-Based Nanoparticles

Because of their exceptional magnetic properties, surface-to-volume ratio perfect for successful functionalization, and biocompatibility, iron oxide NPs are often used in biological applications. Because these nanoparticles are now utilized in the medical system as contrast agents and heating mediators, most of the attention is focused on their development for MRI or magnetic particle hyperthermia. As a result, it is essential to keep improving and making new materials that are better and more reliable [76] for molecular imaging and biosensing.

#### Super Paramagnetic Iron Oxide Nanoparticles for Cancer Treatment

Superparamagnetic iron oxide nanoparticles (SPIONs) have drawn more attention due to their superior superparamagnetism, magnetic heating abilities, and improved magnetic resonance imaging (MRI). In vivo imaging, magnetic thermotherapy, and simultaneous delivery of anticancer treatments are only a few benefits of conjugating SPIONs with medications to create delivery nanosystems. Additional targeting moieties such as transferrin, hyaluronic acid, antibodies, aptamers, folate, and targeting peptides are coated onto the surface of SPIONs to improve the targeting efficacy of pharmaceuticals delivered by a delivery nanosystem based on SPIONs [77]. SPIONs exceptional MRI enhancement capabilities can be employed as tracers to depict the location and status of illness in the body, in addition to being deposited in cancer cells through the EPR effect and under an external magnetic field. Furthermore, SPIONs have a lower potential for toxicity than other inorganic NPs like their carbon- and gold-based counterparts because they can biodegrade into ferric ions in the human body, particularly in cells with acidic conditions (such as the lysosome and endosome) [78]. Theranostic agents based on SPION are essential for the delivery of therapeutic payloads such chemotherapeutic drugs and genes and for the diagnosis of cancer.

### 3.5. Copper-Based Nanoparticles

For biomedical applications, copper-based NPs have gained more interest. When exposed to a near-infrared laser, copper chalcogenide NPs display excellent near-infrared absorption, exhibit effective light-to-heat transformation, and selectively thermally destroy the tumor. Smaller copper NPs demonstrate the fluorescence signal and optical imaging capabilities. Additionally, copper-based NPs provide a flexible means of drug administration and image-guided treatment. Current developments in the biological use of copper-based NPs with an emphasis on cancer imaging and therapy have been discussed by Zhou et al. [79].

Copper NPs have more uses than Au and Ag NPs due to their reduced price, greater cytotoxic action against cancer cells at low doses, and prolonged stability period [80]. Novel copper-containing NIR-absorbing nano-formulations, such as copper selenide (Cu2-xSe) nanocrystals, nanocubes, monodispersed CuTe nanorods, nanoplates, and copper bismuth sulfide (Cu_3_BiS_3_) nanostructures, have all been fabricated and further confirmed for PTT. All the nanomolecules of copper discussed above have strong anticancer potential and improved photothermal heating efficiency [81].

### 3.6. Selenium-Based Nanoparticles

The trace element selenium (Se) is essential. It is included as selenocysteine, the most significant component of the active centre of selenoproteins’ enzymatic activity. Numerous selenoproteins have oxidoreductase activity and hence control the redox balance in the body. Se has a small therapeutic window and very fragile toxicity margins, but Se nanoparticles (SeNPs) have remarkably lower toxicity. SeNPs have been investigated for their potential therapeutic effects in a number of oxidative stress and inflammation-induced diseases, including cancer, diabetes, nephropathy, and arthritis. SeNPs serve as a desirable drug delivery system for a variety of medications. The impact of nanosizing on Se’s pharmacological action has been covered in this article. Presently discussed is the function of SeNPs in the pharmacological defence against diverse inflammatory and oxidative stress-mediated situations. SeNPs’ potential impact on the pharmacokinetics and pharmacodynamics of selenoproteins, however, remains mainly unknown.

Human clinical trials validate the protective and curative functions of selenium in the initiation and progression of cancer. Diverse anticancer processes of selenium [82] can be divided into three major categories: thiol modification, chromatin binding and alteration, and reactive oxygen species (ROS) generation. Important factors affecting selenium’s biological activity, toxicity, and ability to prevent cancer are its amount and form [83]. Selenium’s anticancer activities have traditionally been linked to its organic form, particularly dietary selenium, and its antioxidant and pro-oxidant capabilities.

Studies on several malignancies, such as breast, lung, prostate, and colon cancers, demonstrate cancer-protective effects.

The most prevalent form of selenium in plants, seleniomethionine, was once believed to be the most chemopreventive and therapeutic form of selenium, but more recently, methylselenocysteine was discovered to have higher biological activity [84]. Since then, synthetic organoselenium species have been created that outperform their natural counterparts in terms of anticancer activity [85].

**Figure 4:**
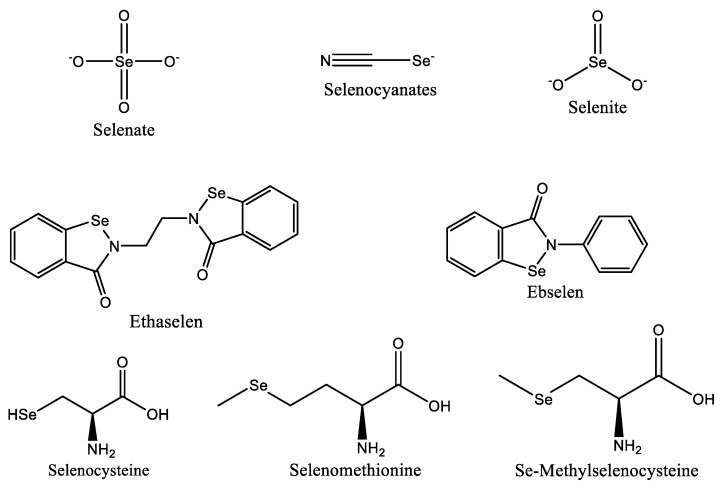
Selenium species with anticancer properties.

Despite their origins as an antioxidant, selenium-based medications like ebselen (Figure 4) have demonstrated potential anticancer action against breast, liver, and colon cancer cells. In Phase I clinical studies for the treatment of non-small cell lung cancer, ethaselen, a modified version of ebselen, has demonstrated better solubility [82,86]. According to studies, selenium can prevent cancer by preventing DNA damage brought on by the production of adducts caused by dimethylbenz(a) anthracene, which is a factor in the development of breast, colon, and liver cancers [87].

## 4. Nanoparticles for Medical Imaging

Medical imaging is commonly used to investigate biological processes, spot anomalies, and track the development of illnesses [88]. The clarity of medical pictures is continually being enhanced with the development of cutting-edge imaging technology. These methods include magnetic resonance imaging (MRI), computed tomography (CT), positron emission tomography (PET), optical fluorescence imaging, photoacoustic imaging (PA), single-photon emission computed tomography (SPECT), and ultrasound imaging (US) [89].

Imaging techniques serve a crucial function in the diagnosis and therapy of cancers. Many NPs, such as iron oxide NPs [90], can enhance imaging due to their magnetic, optical, acoustic, and structural properties [91]. Previous studies reported that injecting NPs into target tissues can improve image guidance and contrast for locating and removing tumors [92]. For instance, in cryosurgery, NPs can enhance the tumor’s and the ice ball’s imaging quality, enabling more accurate coverage and improved therapeutic effectiveness [93]. In addition, the majority of imaging NPs are composed of various metals. Table 2 provides examples of NPs derived from multiple materials and their application in medical imaging [94].

A non-invasive, micron-level resolution and biological imaging method is optical coherence tomography (OCT). Real-time diagnosis and surgical guiding are both facilitated by OCT. However, it cannot pick up inelastic dispersed light because it lacks coherence in the incident field [100]. Numerous studies have recently shown that the motion state of NPs may alter the amplitude of OCT, which could resolve this issue. Interfering with the movement of NPs in the magnetic field causes local changes in the dispersion of light. By adding magnetic NPs, which may alter optical scattering and make it possible to identify originally incoherent inelastic scattered light, one can regulate the movement of a magnetic field. The name of this cutting-edge imaging technique is magnetomotive optical coherence tomography (MMOCT) [96].

Magnetic resonance imaging (MRI) is one of the most effective non-invasive tumor detection methods [101]. However, the absence of an MRI signal comparison between normal and cancerous tissue hinders the clinical tumor diagnosis [102]. MRI is a scanning imaging technique that assesses the degree of hydrogen molecule magnetization inside water molecules. Due to the distinct variations in magnetization caused by each tissue’s protons, each anatomical structure shows a unique picture. Applying more excellent contrast agents can increase the visibility of images [103]. MRI scanning is a beneficial medical diagnostic technique for an accurate anatomical picture, and for increased diagnostic sensitivity, contrast agents are frequently utilized in MRI [104]. Using MRI, one can spot bodily tissues, organ states, blood flow, and physiochemical characteristics [105]. Chelate-based traditional contrast agents have limitations due to their biological stability and degree of toxicity when accumulated in cells [106]. For example, iodine is present in several contrast agents. The occurrence of incident hyperthyroidism has been associated with exposure to iodinated contrast media [107]. Instead of using contrast chemicals, which may be damaging to the body, alternatives have been developed to improve the scanning efficiency [108]. There are also metal NPs that can be combined with a material to work similarly to a contrast agent for MRI scanning [109].

Nanoparticles possess unique optical and magnetic properties that can be utilized to improve imaging techniques. For example, quantum dots are semiconductor nanoparticles that emit light of different wavelengths based on their size, making them ideal for fluorescent imaging. Iron oxide nanoparticles can be used as contrast agents in magnetic resonance imaging (MRI) due to their magnetic properties, providing better visualization of tumors. Gold nanoparticles, on the other hand, exhibit strong absorption and scattering of light, enabling enhanced photoacoustic imaging. These nanoparticles enhance the sensitivity, resolution, and specificity of imaging, allowing for early cancer detection, precise tumor localization, and monitoring of treatment response.

## 5. Targeting of Cancer Cells by Metallic Nanoparticles

An effective method for cancer diagnosis and treatment is to target cancer cells using metallic nanoparticles. Recently published research on the topic includes the following:

*1. Copper nanoparticles*: Copper nanoparticles have recently received interest as potential anticancer therapies due to their ability to induce oxidative stress in cancer cells. Reactive oxygen species (ROS), which lead to cell death, are produced by copper nanoparticles, which have been demonstrated to selectively target cancer cells while sparing healthy cells [110]. Additionally, it has been shown that copper nanoparticles increase the responsiveness of cancer cells to radiation therapy, promoting tumor remission [111].

*2. Silver nanoparticles*: Silver nanoparticles have also been investigated as a potential cancer therapy. By interacting with proteins on the cell surface and causing cell death by creating ROS, researchers have shown that silver nanoparticles may selectively target cancer cells [112]. Silver nanoparticles have also been studied as imaging agents for cancer diagnosis due to their powerful optical properties [113].

*3. Zinc oxide nanoparticles:* These particles have been studied as potential anticancer therapies since they may induce cancer cells to undergo apoptosis. The ability of zinc oxide nanoparticles to precisely target cancer cells and induce cell death by triggering caspases and suppressing anti-apoptotic proteins has been established [114]. Additionally, zinc oxide nanoparticles have been shown to overcome the drug resistance of cancer cells, improving the efficacy of chemotherapy treatments.

Recent research on metallic nanoparticles for cancer targeting has generally focused on improving the specificity and efficacy of existing agents as well as looking into novel kinds of nanoparticles with unique properties for cancer diagnosis and therapy.

A surplus of molecular information has resulted from the quick advancement in our understanding of the molecular pathophysiology of illnesses and the development of novel molecular biology tools. The speed of these advancements has led to the identification of numerous molecular targets for pharmacological action at a rate that much exceeds our current capacity to use this molecular knowledge [115,116]. Research and development efforts are currently being made to find and create medications that disrupt the signal transduction pathways used only by cancer cells. These medications will facilitate doctors to customize each patient’s care based on the specific molecular targets that their tumor produces. Drugs can be administered directly to the target organs where the tumor is located or to the cancer cell’s surface. They can also be administered using a drug delivery system. The primary components of such a targeted drug delivery are stated in the following:

- The availability of specific targets first bullet;
- Ligands for these targets;
- Techniques for delivering the drug to its target via various delivery systems conjugated to the ligands.

Each form of cancer requires a distinct approach since they might be solid tumors or hematologic malignancies. Drug administration is made more difficult by solid tumors’ heterogeneous and dynamic biology, which constantly changes over time [117]. A complete understanding of the biology of tumor cells, their microenvironment, and their development patterns enables the creation of efficient, targeted drug delivery systems.

**Figure 5:**
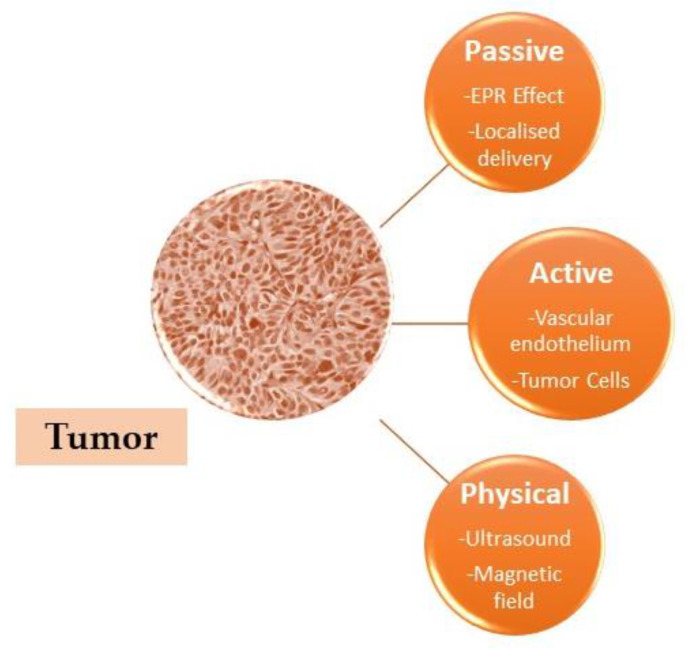
Several drug-targeting techniques for the tumor.

A drug can be targeted in two ways: by actively pursuing a drug carrier using target-specific ligands, or by using the distinct pathophysiological features of tumor tissue (Figure 5) [118].

Development or engineering of a medication or gene delivery system with an outstanding capacity to target tumor cells while preserving the normal healthy cells is vital for effective cancer therapy. It increases therapeutic effectiveness, protecting healthy cells from cytotoxicity. It can be done by strategically delivering NPs to the tumor microenvironment (TME) and then indirectly targeting the cancer cells there. These nano-formulations should be able to transit through a number of pharmacological and biological barriers. These barriers are intricate systems made up of cellular membranes, mechanical and physicochemical barriers, enzymatic barriers, and layers of epithelium, endothelium, and other tissue. To avoid non-specific targeting, these realities restrict the biocompatibility, surface chemistry, and size of NPs. However, merely internalizing an NP drug molecule in the cytosol does not guarantee that it has reached its subcellular target. To allow cellular or nuclear targeting, specific engineering and optimization are required [119].

### 5.1. Targeting Cancer Stem Cells with Nanomaterials

**Figure 6:**
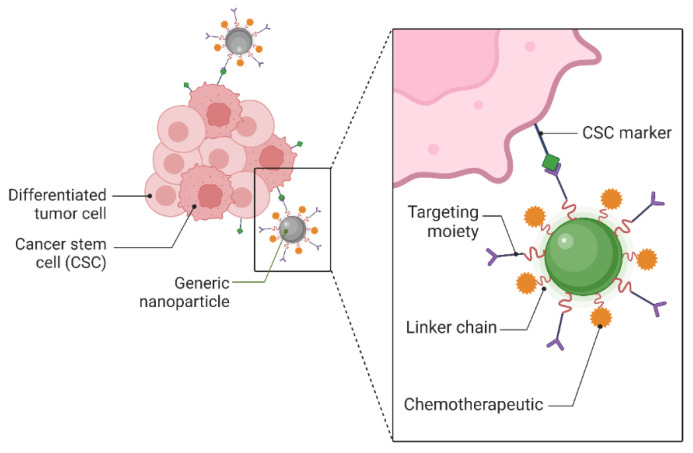
Active targeting of cancer stem cells with nanoparticles.

A collection of proliferating cells with high power and excellent resistance to medications, known as cancer stem cells (CSCs), is the primary cause of cancer. In the 1990s, CSCs were discovered and defined in the blood of leukaemia patients, and they appeared to play a significant role in malignancy. The CSCs promote tumor development, resist traditional treatments (such as chemotherapy and radiation), cause disease recurrence, and produce metastases. Consequently, there is a lot of interest in using nanoscale materials for CSC-directed cancer therapy. The NP surface has been engineered to precisely and successfully target the CSCs [120]. Potential therapeutic targets for eradicating cancer stem cells (CSCs) are surface indicators unique to CSCs. By joining targeting molecules to drug-delivery NPs, active targeting can be accomplished. These molecules can only attach to the markers present on CSCs. After binding, both internal and external events might cause drug release. These techniques have been used to create a variety of NP-based drug delivery platforms, as shown in Figure 6 [121].

Chemotherapy and radiation are two standard cancer therapies that CSCs notably resist. Therefore, to create effective medicines, knowing tumor biology is essential. New therapeutic approaches for cancer treatment include identifying and specifically targeting CSC-specific markers and signalling pathways [122,123,124]. Studies utilizing nanotechnology-based therapies with several surface markers and biochemical tests for identification have revealed the present effectiveness in the battle against CSCs [125].

### 5.2. Targeted Drug Delivery

**Figure 7:**
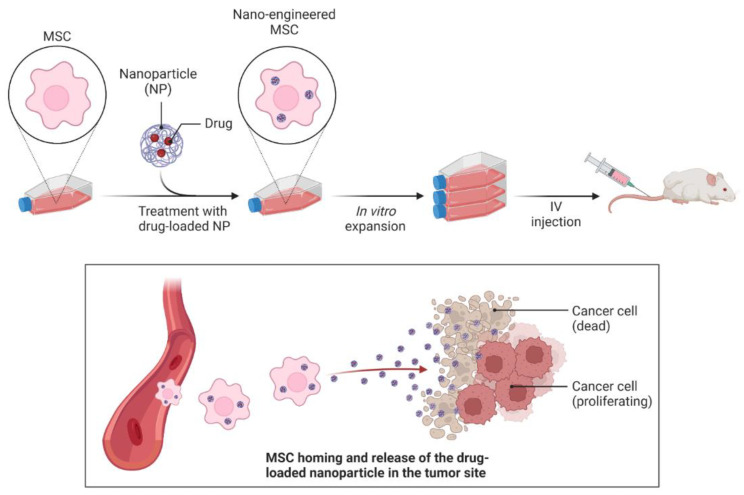
Nano-engineered MSCs as active targeting drug delivery vehicles.

Despite being the most popular form of cancer treatment today, chemotherapy still suffers from the issues of inadequate target enrichment in regions of cancerous tumors and over-accumulation in healthy tissue [126]. Inhibiting cells that reproduce rapidly, such as hair follicles, bone marrow, gastrointestinal cells, and lymphocytes, may result in undesirable side effects like hair loss, mucositis, and even death. The use of inorganic nanoparticles in gene and drug therapy opens the door to targeted cancer detection and therapy with enhanced efficiency and better treatment outcome [85]. Compared to the standard of care, targeted medication delivery, which involves actively differentiating between healthy and cancer cells, is more effective and causes fewer side effects. Numerous studies have demonstrated that NPs may actively or passively target chemotherapy medicines to tumor cells [127]. Additionally, in several studies, NPs have been revealed to be crucial for the targeted administration of immunological medications [128]. The lack of cancer cell selectivity in chemotherapeutic drugs and the formation of many undesirable side effects prompted the development of innovative drug delivery techniques. The growth of “nano-engineered” mesenchymal stem cells (MSC) that can actively target the tumor site and shield the drug-loaded NP from vascular filtration and macrophage clearance is made possible by combining nanotechnology with cell therapy (Figure 7) [129,130].

Targeted medication delivery to tumors has the potential to lower peripheral/systemic toxicity, enhance the selectivity for killing cancer cells, and allow for dosage escalation. With improvements in identifying tumor-specific targets and developing several drug delivery methods for tumor targeting, hopes for developing an efficient, targeted drug delivery modality for cancer therapy have grown. Although the ultimate goal is to remove cancer from the patient altogether, more pragmatic aims to enhance the patient’s quality of life are near to being realized. In the subsequent years, the focus will be on creating systems that can effectively internalize into cancer cells while also being able to detect specific targets on cancer cells. Some of these issues could be solved by combining targeted strategies. Other promising ideas for medication targeting in cancer therapy include employing unique molecular addresses on the vascular endothelium and targeting using magnetic fields and ultrasound.

Nanoparticles can be engineered to selectively target cancer cells while sparing healthy tissues. This is achieved by attaching specific targeting ligands, such as antibodies or peptides, onto the nanoparticle surface. These ligands can recognize and bind to specific receptors or biomarkers overexpressed on cancer cells, facilitating the preferential accumulation of nanoparticles at the tumor site. By encapsulating or conjugating therapeutic agents, such as chemotherapy drugs, small interfering RNA (siRNA), or gene therapy vectors, nanoparticles can deliver these payloads directly to cancer cells. This targeted approach increases drug concentration within the tumor, reducing off-target effects and minimizing systemic toxicity.

### 5.3. Metal Nanoparticle Mediated Cryosurgery

Benefits of cryosurgery, the freezing of tumor tissue, include minimal cost, less invasiveness, decreased intraoperative bleeding, and fewer postoperative issues. Still, drawbacks include poor freezing efficiency and freezing damage to nearby tissues [93]. Antifreeze protein (AFP-1) has been used as a protective factor to aid with cold ablation, although the results are still not perfect [131]. As nanotechnology evolved, nano-cryosurgery arose as a concept. Nano-cryosurgery is predicated on the delivery of NPs with precise physical or chemical characteristics into tumor tissues. By utilizing the properties of NPs, it is able to manage range modification, ice ball formation direction, and freezing efficiency. As a result, nano-cryosurgery can destroy tumor tissue while shielding nearby healthy tissue from freezing simultaneously [132].

Intracellular ice creation during cryosurgery is essential for tumor cell destruction. Researchers have shown that NPs can successfully promote the development of intracellular ice in the interim [108]. NPs can cause heterogeneous nucleation by acting as outside particles. According to a recent study, NP-enriched tissues freeze more quickly than unenriched tissues and are more prone to heterogeneous nucleation. Moreover, NPs can considerably speed up and enhance the likelihood of ice production in cells, killing tumor cells more efficiently [133]. It has been demonstrated by the ease with which ice forms in tissue when the same freezing conditions are applied. Additionally, the thermal conductivity in tumor tissue would be greatly increased by NPs containing a metal oxide.

Since tumors are typically shaped irregularly, the ice crystals created by conventional cryosurgery only partially cover the tumor tissue. Nano-cryosurgery is more effective than traditional cryosurgery at solving this issue. Due to the fact that NPs may enter intracellular fluid and possess favourable physical characteristics, such as thermal conductivity, it is feasible to regulate the direction of ice ball development by scattering them [112]. Healthy tissue is more susceptible to injury. In recent years, cryosurgery utilizing phase change materials (PCMs) generated by nanoparticles has proven to have considerable promise for protecting the surrounding healthy tissues [134]. The use of NPs in cold ablation falls broadly into two categories: synergistic effect and protective effect, which differ in the NPs design specifications and in vivo dispersion. Future NPs may help nano-cryosurgery by being disseminated around the tumor while protective NPs are spread inside the cancer. In addition to overcoming the challenges of cold ablation of rare tumors, nano-cryosurgery technology can also improve the effectiveness of cold ablation and lessen the harm to good tissue. Table 3 lists a few recent instances of NPs being employed in cryosurgery [94].

## 6. Types and Biological Properties of Nanoparticle Delivery Platforms

Using molecular platforms created for drug transport and selective targeting could be more practical. Lipid nanocarriers (micelles, liposomes, and lipid nanoparticles), polymer conjugates, polymeric nanoparticles, and antibody-drug conjugates are a few examples of the proposed molecular platforms (ADCs). Selective targeting uses a platform’s inherent properties like size, charge, or the presence of a particular polymeric component (such as a polymer recognized by a specific receptor). It is also possible to directly functionalize a molecular platform’s surface with a targeting agent or moiety.

While natural and synthetic polymers have found use in various industries, from manufacturing materials to medicinal uses, recent decades have increased interest in using them as parts of complex drug delivery systems. Due to these advancements, a brand-new class of medications known as “polymer therapies” [139] emerged. Polymer-drug conjugates, polymer-DNA complexes (or polyplexes), polymer-protein conjugates, polymeric micelles, and polymer-aptamer conjugates, containing medicines covalently tethered to the polymer carrier, are some examples of these novel chemical entities [140,141].

**Figure 8:**
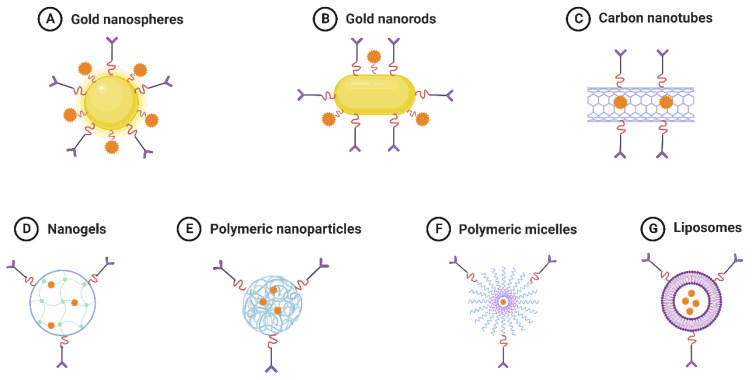
Drug delivery platforms for cancer treatment. (A) Gold nanospheres, (B) Gold nanorods, (C) Carbon nanotubes, (D) Nanogels, (E) Polymeric NPs, (F) Polymeric micelles, and (G) Liposomes.

Traditional micelles and liposomes, which depend on the physical encapsulation of medicines, can have problems, including the early release of the active ingredient, instability during storage and administration, and poor encapsulation. An early medication release impairs the effectiveness of treatment and causes systemic toxicity. Since pharmaceuticals are conjugated to the polymeric backbone utilizing cleavable linkers that only trigger drug release under specific circumstances, polymer treatments can address these problems [142]. A range of medication delivery methods for the treatment of cancer is shown in Figure 8 [143].

## 7. Current Limitations of Metal Nanoparticles and the Challenges

The use of metal nanoparticles in detecting and treating cancer comes with several limitations and challenges, despite the potential benefits. Following are some existing limitations and challenges mentioned in this section:

*Biodistribution and toxicity*: One of the main problems with using metal nanoparticles is their potential toxicity and biodistribution in the body. According to certain research, metal nanoparticles can accumulate in organs and tissues and cause toxicity and adverse effects. It is essential to accurately evaluate the toxicity and biodistribution of metal nanoparticles before using them in therapeutic contexts [144]. Due to their extremely small physical dimensions, nanoparticles are more hazardous than larger particles. Although fullerene and carbon nanotubes are highly hazardous when inhaled into the lungs, carbon black is not toxic. Similar to this, titanium oxide nanoparticles have been demonstrated to increase toxicity and cause oxidative stress in bacterial cells. Many non-toxic bulk substances turn poisonous when scaled down to the nanoscale. It is crucial to accurately assess the toxicity and biodistribution of metal nanoparticles before using them in therapeutic contexts.

*Stability and aggregation*: Metal NPs’ instability and susceptibility to aggregation may limit their effectiveness and selectivity for fighting cancer. Aggregation or agglomeration can take place in metal nanoparticles, which can result in modifications to the nanoparticles’ physicochemical characteristics and a decrease in their therapeutic efficacy. Several environmental conditions, such as changes in pH or the presence of biomolecules, can cause aggregation in biological systems. Metal nanoparticles must be stabilized and prevented from aggregating in order to be used in clinical settings with confidence. Researchers have recently devised unique techniques to maintain the stability of metal nanoparticles and prevent aggregation, including using surface coatings and conjugation with specific ligands [145].

*Specificity and selectivity*: In the process of using metal nanoparticles to target cancer, one of the challenges is attaining a specificity and selectivity adequate for cancer cells while sparing normal cells. Traditionally, cancer-treating chemotherapeutic agents are distributed non-specifically, damaging both healthy tissue and cancerous ones, resulting in low efficacy and high toxicity. Controlled drug delivery systems would be excellent carriers for chemotherapeutic agents, directing the chemotherapeutic agents to the tumor site, thereby increasing drug concentration in cancer cells and preventing toxicity to normal cells. Researchers are investigating new targeting ligands and techniques to boost the specificity and selectivity of metal nanoparticles for cancer cells [146].

*Regulatory approval:* The procedure for obtaining regulatory permission for the use of metal nanoparticles in therapeutic applications may be difficult and time-consuming. Extensive preclinical and clinical research is required in order to evaluate their safety, effectiveness, and impacts over the long term. In order to successfully incorporate metal nanoparticles into cancer diagnosis and therapy, it is vital to close the gap that exists between research conducted in the laboratory and its use in clinical settings. The clinical translation of metal nanoparticles for cancer diagnosis and treatment requires regulatory approval and clinical investigations. This strategy calls for substantial preclinical and clinical testing, which may be time-consuming and expensive. Still, it is necessary to guarantee that the treatment will be safe and effective.

To address these limitations and challenges, researchers, engineers, healthcare providers, and regulatory bodies must collaborate across disciplines. To overcome these obstacles and realize their full potential for cancer treatment and other biomedical applications, continued research and development in the field of metal nanoparticles is required.

## 8. Conclusions and Future Perspective

Biomedical applications of metal NPs have been the subject of many studies. They are becoming increasingly popular in the biomedical sector due to their high inertness and nanoscale architectures, which are equivalent in size to many biological molecules. Particle intrinsic features, such as electrical, physicochemical, and optical surface plasmon resonance, can be altered by modifying particle characteristics, including size, ease of synthesis, shape, environment, aspect ratio, and functionalization qualities. Numerous applications in a range of biomedical fields have resulted from this. Targeted medication delivery, sensing, imaging, photothermal and photodynamic therapy, and controlling two or three applications are a few of these applications.

Cancer is one of the diseases with the highest mortality rate in the world, even though there are already treatments available for cancer that have considerable drawbacks. Therefore, new anticancer strategies are once again necessary. One of the most promising treatments for cancer treatment is metal NPs. Iron, gold, silver, zinc, and titanium are examples of metals that may function as anticancer agents, naturally or by surface modification. Metal NPs are essential therapeutic and diagnostic tools in the fight against cancer.

According to researchers working to improve clinical outcomes, the greatest obstacle is chemotherapy for cancer. The therapeutic efficiency of cancer treatments may be enhanced with tailored medication administration, while unfavourable side effects may be decreased. Nanotechnology has caused a fundamental shift in medical science. One of the upcoming generation’s promising anticancer drugs is metal NPs. The extraordinary physical and chemical flexibility of metal nanoparticles has attracted a lot of research. Due to their distinctive plasmonic properties, noble metal NPs also offer a reliable way to monitor nano-complex drug carriers inside the body, making them a more effective treatment option with a reduced risk of adverse effects than conventional treatments. By using these NPs, practitioners may learn about and track therapeutic outcomes during therapy. Non-noble metal NPs are inexpensive and have distinct qualities, including magnetic and thermal capabilities. Metal nanoparticles have shown promise as a cancer treatment in several studies, and different compositions are now undergoing preclinical and clinical testing. With NP-based imaging, it may be possible to pinpoint the stage of a tumor, and approaches for treating it may be developed that reduce or eliminate the expected toxicity levels. Before conducting clinical research, however, it is necessary to analyze many production and utilization traits. Some measures include controlling preparation methods, reproducibility, stability, dose, the amount of accumulation at target and off-target areas, and, most crucially, toxicological concerns.

The future perspectives of nanoparticles in cancer diagnosis and treatment are highly promising. Following are some potential areas where nanoparticles will have a significant impact:

Early cancer detection: NPs can be engineered to specifically target biomarkers associated with cancer cells, allowing for highly sensitive and specific early detection. This could enable the diagnosis of cancer at its earliest stage, when treatment is most effective.

Improved imaging techniques: NPs can enhance existing imaging modalities such as MRI, CT, and optical imaging. By functionalizing nanoparticles with targeting ligands and contrast agents, they can improve the resolution, sensitivity, and specificity of cancer imaging, enabling accurate tumor localization and monitoring of treatment response.

Targeted drug delivery: NPs can serve as vehicles for targeted drug delivery, improving efficacy and reducing the side effects of chemotherapy. They can encapsulate anticancer drugs and deliver them directly to tumor sites, minimizing damage to healthy tissues. Additionally, stimuli-responsive nanoparticles can release drugs in response to specific triggers, such as pH, temperature, or enzyme activity within the tumor microenvironment.

Combination therapy: NPs offer opportunities for combining multiple therapeutic modalities into a single system. For example, nanoparticles can be loaded with chemotherapy drugs, immunotherapeutic agents, or gene therapies, allowing for synergistic effects and personalized treatment approaches. This approach holds promise for overcoming drug resistance and improving overall treatment outcomes.

Theranostics: NPs can integrate both diagnostic and therapeutic functionalities into a single system, known as theranostic nanoparticles. These multifunctional nanoparticles can simultaneously diagnose cancer, deliver therapy, and monitor treatment response. They have the potential to revolutionize personalized medicine and enable real-time monitoring of treatment efficacy.

Immunotherapy enhancement: NPs can play a crucial role in enhancing the efficacy of immunotherapies, such as immune checkpoint inhibitors or cancer vaccines. They can be used to deliver immunomodulatory agents, antigens, or adjuvants directly to immune cells or tumor sites, stimulating a robust immune response against cancer cells.

Microenvironment modulation: NPs can be designed to target the tumor microenvironment and modify its characteristics. They can normalize abnormal blood vessels, enhance drug penetration into tumors, modulate immune responses, or inhibit metastasis, thus improving the overall treatment outcomes.

Regulatory bodies play a crucial role in developing new clinical criteria for using metal NPs in cancer therapy and drug delivery, as well as new tools for evaluating the effectiveness and safety of such nanoparticles. Furthermore, scientists are striving to identify the best phytochemical conjugated metal NPs dosages for cancer patients and the most efficient way to give these doses. Once these issues are resolved, it is conceivable that metal NPs will become an indispensable therapeutic weapon in the fight against cancer.

There is an increasing demand for the practical translation of these technologies due to the continual advancement of research in nanoparticles and cancer. Researchers are attempting to create nanoparticle-based therapeutics that are both safe and effective so that they may be utilized in medical facilities to improve the results of patients who have cancer.

## Figures and Tables

**Table 1:** Summary of Pd-based nanoparticles for cancer treatment.

Type of Therapy	Material	Reference
Photothermal therapy (PTT)	Pd NSs	[55,56]
	Pd collora	[57]
	Pd@Ag nanoplates	[58]
	Pd@Au nanoplates	[59]
	Pd NS-CO Pd-TAT	[60]
PTT and photodynamic therapy (PDT)	Pd@Pt-PEG-Ce6	[61]
	Pd@Ag@mSiO2-Ce6	[62]
	Pd-PEI-Ce6	[63]
	H-Pd NSs	[64]
	PLCs-HSA-ICG	[65]
PTT and chemotherapy	Dox-loaded 8dc-Pd NPs	[66]
	SPNS-DOX	[67]
	Pd@Au-PEG-Pt	[68]
	HMSS-NH2/DOX@Pd	[69]
PTT and radiation therapy	[131I]PHPdNPs-DOX	[70]
	131I-Pd-PEG	[71,72]
PTT and immunotherapy	Pd-CpG	[73]
PTT and hydrogen therapy	PdH0.2 nanocubes	[74]
	PdH-MOF	[75]

**Table 2:** Different materials make typical NPs platforms for medical imaging.

Nanoparticles	Size (nm)	Targeting Material	Cell Line	Imaging Technology	Ref.
PLGA-mPEG	151.1 ± 1.3	cRGD	SKOV-3 cells	Ultrasound	[95]
MnO-TETT	6.7 ± 1.2	None	C6 glioma cells	Fluorescence/T1-MRI ^1^	[96]
Ultra small MnO@mesoporous silica	30–50	Dox	HeLa cells	MRI-guided chemotherapy	[97]
PEG–coated and Gdloaded flourescent silica	125.5 ± 9.9	YPSMA-1	LNCaP and PC3 prostate cancer cells	MRI/fluorescence imaging	[98]
Oxygen/indocyanine green-loaded (OINPs)	300	Folate	SKOV3 ovarian cancer cells	Ultrasound/ photoacoustic	[99]

^1^ Magnetic resonance imaging.

**Table 3:** Common nanoparticle platforms utilized in cryosurgery.

NPs	Size (nm)	Thermal Conductivity (W/m K)	Heat Capacity (J/m^3^ K)	Benefits	Ref.
Fe_3_O_4_	8–14	7.1	3.2 × 10^6^	More intracellular ice formation, high thermal conductivity	[135]
MgO	50	34.3	3.2 × 10^6^	Nontoxic, biodegradable, and few side-effects	[133]
HCPN-CG	103.9 ± 1.5	None	None	Cold-responsive NP for control drug release and NIR-induced photothermal effect.	[136]
PCM	10–20	0.35	2.56 × 10^6^	Health tissue protection	[137]
Au	3	297.7	2.2 × 10^6^	Good biological compatibility, high thermal conductivity	[138]

## Data Availability

Not applicable.
